# Activity of Superoxide Dismutase (SOD) Enzyme in the Excretory-Secretory Products of *Fasciola hepatica* and *F. gigantica* Parasites

**Published:** 2013

**Authors:** A Farahnak, A Golestani, MR Eshraghian

**Affiliations:** 1 Department of Parasitology and Mycology, School of Public Health, Tehran University of Medical Sciences, Tehran, Iran; 2 Department of Biochemistry, School of Medicine, Tehran University of Medical Sciences, Tehran, Iran; 3 Department of Epidemiology and Biostatistics, School of Public Health, Tehran University of Medical Sciences, Tehran, Iran

**Keywords:** *Fasciola*, Superoxide Dismutase, Excretory-Secretory, Helminth

## Abstract

**Background:**

The aim of this study was to compare superoxide dismutase (SOD) activity in *Fasciola hepatica* and *Fasciola gigantica* parasites.

**Materials:**

*F. gigantica* and *F. hepatica* helminths were collected from abattoir and cultured in buffer media for 4 h at 37 °C. Excretory-Secretory (ES) products were collected, centrifuged and stored at -20^°^C. E-S protein concentration was measured by Bradford method and SOD activity was detected using RANSOD kit (Randox Lab. Crumlin, UK). Statistical *t*-test was conducted for analysis of results.

**Results:**

Protein concentration for *F. hepatica* and *F. gigantica* were obtained 7.293 ug/ml and 19.65 ug/ml respectively and SOD activity as 0.721 U/ml and 1.189 U/ml, in that order. ES protein concentration of two species was significantly different (*P*<0.05), however the difference of SOD activity of two species was not significant.

**Conclusion:**

Two species of *Fasciola* have comparable SOD biochemical defense enzyme and can help us explain the parasite survival in host tissue.

## Introduction

Fascioliasis, an infection caused by *Fasciola hepatica* and *Fasciola gigantica*, has been considered as an important veterinary disease. In contrast, human fascioliasis has been regarded as a secondary disease (1). Oxygen can be converted to form extremely reactive radicals that bind to DNA, proteins and lipids causing permanent loss of structure to such biomolecules. The cells have superoxide dismutase (SOD) enzymes to protect them from reactive radicals. SOD, is an enzyme whose active center is engaged by copper, zinc, manganese or iron. SOD is an enzyme that plays major roles in the protection of cells against oxidative damage (2). SOD has been determined in excretion-secretion (ES) of *F. hepatica* using the xanthine oxidase system and detected in substrate gels (3).

Apparently there is no study to compare SOD enzyme activity in the ES of *Fasciola* species in Iran. Comparison of SOD activity in two species of *Fasciola* improves our understandings on the impact of species on biochemical defense mechanism of these parasites.

For these reseans, enzyme activity assay approach was used for comparative analysis of SOD enzyme activity of ES products from *F. hepatica* and *Fasciola gigantica* parasites.

## Material and Methods

### ES Products Collection

Infected sheep livers with *Fasciola* spp parasites were gathered from abattoirs on the day of slaughter and were transferred to helminthological lab, School of Public Health, Tehran University of Medical Sciences, Iran. The livers were dissected with the scalpel and the adult parasites as alive were collected from hepatic bile ducts. *F. hepatica* and *F. gigantic*a parasites were identified based on morphological (cone and end parts characterizations) and morphometric (length size) parameters. Collected *Fasciola* spp. parasites were washed for a minimum of three times in PBS, pH 7.4, to remove host material. Ten *F. hepatica* and 10 *F. gigantica* parasites were cultured in RPMI media for 4 h at 37 °C (one parasite in 2 ml of culture media for each). The ES products were clarified via centrifugation at 4000g for 20 min at 4 °C (4). The ES supernatant was concentrated by Sephadex G-25 (5). A volume of 400 µl of ES products from each 2 ml supernatant was collected and stored at -20 °C until use.

### Protein measurement

Protein concentration of ES products was measured by Bradford method with BSA standard solutions as duplicate (5).

### Enzyme activity assay

**Fig. 1:**
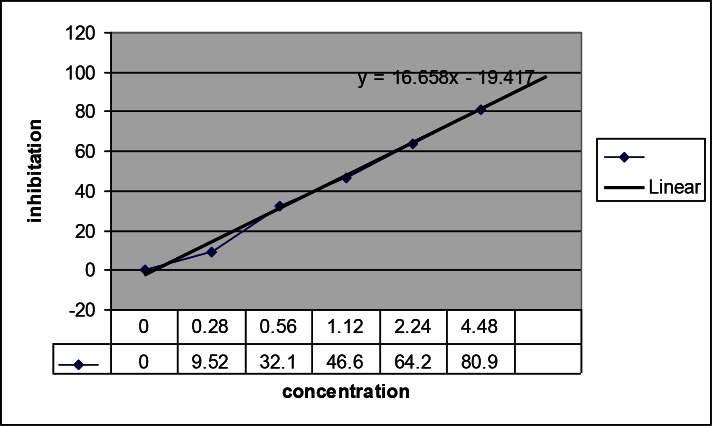
Standard curve using calculated inhibition percent and concentrations of standard solution (SOD enzyme) to determine SOD activity of *Fasciola* samples

SOD activity of ES samples was determined using RANSOD kit (Randox Labs, crumlin, UK) (6). *Fasciola* spp. ES products and standard solutions were used for the assay of SOD. Absorbance was measured at 505 nm on a Cecil 1021 UV / Visible spectrophotometer (Cecil Instruments Ltd Milton Technical Centre Cambridge ENGLAND) for 30 s after the addition of xanthine oxidase to uninhibited, inhibited and standards tubes and *Fasciola* samples to inhibited tubes as start reagent and 3 minute after reaction (Table 1). Inhibited percents of standards and parasite samples were calculated according to superoxide dismutase manual of RANSOD kit and inhibition percent curve was prepared (Fig. 1). The SOD activity value for each sample was read from this curve and SOD activity was expressed as U/ml. One unit is the amount of SOD that inhibits the rate of formazan dye formation by 50%.

**Table 1:** Assay method of SOD activity of standard solutions and parasites samples

Solutions	Inhibited tube	Uninhibited tube	Standard tubes (St_1-5_)
Substrates	(850 µl)	(850 µl)	(850 µl)
Phosphate buffer,7.0	-	(25 µl)	-
Xanthine Oxidase	(125 µl)	(125 µl)	(125) µl
Samples (*F. hepatca., F. gigantica*)	(25 µl)	-	-
Standard solutions 0.2, 0.5, 1.0, 2 and 4 U/ml	-	-	(25 µl)

### Statistical analysis

To determine the statistical difference between protein concentration and SOD enzyme activity samples mean of *F. hepatica* and *F. gigantica*, two-sample (independent) *t*-test was conducted.

## Results

The levels of protein concentration for the parasites’ ES products were obtained 7.29 ±4.161ug/ml and 19.65±4.151ug/ml respectively. Total SOD activity and SOD specific activity in solutions were calculated, and the results presented in Tables 2, 3.

**Table 2:** Absorbance measures, calculated inhibition percent and total activity of SOD enzyme in standard solutions

A1 *	A2	A2-A1	A2-A1/3	**Calculated inhibition percents of standards	***Concentration of SOD in standards(U/ml)
0.118	0.204	0.086	0.028	0	0
0.131	0.147	0.016	0.005	80.9	4.48
0.120	0.150	0.030	0.010	64.2	2.24
0.141	0.185	0.044	0.014	47.6	1.12
0.130	0.187	0.057	0.019	32.1	0.56
0.146	0.222	0.076	0.025	9.25	0.28

* A (Absorbance) was measured at 505 nm on UV / Visible spectrophotometer (Absorbance 1 and 2; first and second absorbances of samples)/

** Inhibition percents of standards was calculated according to superoxide dismutase manual of Ransod Kit

*** Various concentrations of SOD were prepared according to superoxide dismutase manual of Ransod Kit

**Table 3:** Absorbance measures (mean of 3 samples), calculated inhibition percent, total activity and specific activity of SOD enzyme in samples

Sample tubes	*A1	A2	A2-A1	A2-A1/3	**Calculated inhibition percent	***Total activity of SOD(U/ml)	****Specific activity (U/mg protein)
Un inhibited	0.118	0.204	0.086	0.028	0	0	0
*F.hepatica* product	0.110	0.171	0.061	0.020	28.57	0.721	0.098
*F.gigantica* product	0.105	0.149	0.044	0.014	50	1.189	0.060

* A (Absorbance 1 and 2; first and second absorbances of samples) was measured at 505 nm on UV / Visible spectrophotometer

** Calculated inhibition percents of samples according to superoxide dismutase manual of Ransod Kit

*** One unit of SOD inhibits the rate of increase in absorbance at 550 nm by 50% under the conditions of the assay.

**** Specific activity is total activity of SOD per milligram of total protein.

There was no significant difference between SOD enzyme activity of *F. hepatica* (M= 0.721, SD= 0.253) and *F. gigantica* (M = 1.189, SD = 0.774) conditions; *t* [4] = 2.7, *P* > 0.05. T-value was 0.995. However there was significant difference for protein concentration of *F. hepatica* (M= 7.293, SD = 4.161) and *F. gigantica* (M= 19.56, SD = 4.151) conditions; *t* [4] =2.7, *P*<0.05. T-value was 3.641.

## Discussion

Proteomics analysis has shown the presence of glutathione transferase, fatty acid-binding protein, actin, glycolytic enzymes enolase, cathepsin L, glyceraldehyde-3-phosphate dehydrogenase and sod enzyme in excretory-secretory of *F. hepatica* (7, 8). Piacenza L, has indicated the presence of superoxide dismutase (SOD) activity in the ES of *F. gigantic*, 7.16 U/mg, and *F.hepatica*, 25 U/mg (9). Our results showed low SOD activity which may result from none purified fraction of parasite ES. ES materials from *F. gigantica* suppress the release of oxidants by activated sheep neutrophils. Fractionation of this ES product on Sephadex G-25 has given two peaks of anti-oxidant activity, one greater than 10 kDa, the other less than 10 kDa (10). In this research one band protein was detected in the sample of not fractionation ES product of both species. In the present work, high concentration of protein in the ES of *F. gigantica* has not shown direct association with SOD enzyme activity and this may be related to the raise of the other proteins. Although, the results of this research for SOD enzyme activity in sheep liver fluke are reasonable, however, another study must be designed to determine enzyme activity in bovine fascioliasis.

## Conclusion

Two species of *F. hepatica and F. gigantica* have comparable SOD enzymes activity as part of biochemical defense mechanism. These findings can also help us explain the parasite survival in host tissue.
